# Actinic keratosis metrics

**DOI:** 10.1007/s00403-024-03305-5

**Published:** 2024-08-20

**Authors:** Sarah E. Burstein, Howard Maibach

**Affiliations:** 1 Brody School of Medicine, East Carolina University, Greenville, NC USA; 2 University of California San Francisco, San Francisco, CA USA

**Keywords:** General dermatology, Medical dermatology, Keratosis, actinic, Observer variation, Reproducibility of results

## Abstract

Actinic keratosis (AK) is a common precancerous skin condition predominantly affecting older males with fair skin and significant UV exposure. The clinical significance of AK is related to its potential for malignant transformation and progression to squamous cell carcinoma (SCC). Accurate diagnosis of AK is essential for adequate treatment, evaluation of therapeutic efficacy, and mitigating the risk of developing SCC. However, clinician variability due to the subjective nature of current diagnostic tools presents significant challenges to achieving consistent and reliable AK diagnoses. Thus, there is no universally accepted standard for measuring AK.

This review evaluates current methods for evaluating and diagnosing AK, focusing on clinician variability through inter- and intraobserver agreement. Eight peer-reviewed studies investigating the reliability of various approaches for AK evaluation show substantial variability in interobserver or intraobserver agreement, with most methods demonstrating only slight to moderate reliability. Some suggest that consensus discussions and simplified rating scales can modestly improve diagnostic reliability. However, remaining variability and the lack of a universally accepted standard for measuring AK underscore the need for more robust and standardized diagnostic and evaluation methods.

The review emphasizes the need for improved diagnostic tools and standardized methods to enhance the accuracy and reliability of AK assessments. It also proposes applying a novel examination approach using 1,3-dihydroxyacetone (DHA) staining which may improve the visualization and identification of AK lesions. Advancements in these areas have significant potential, promising better clinical practices and patient outcomes in AK management.

## Introduction

Actinic keratosis (AK), a common precancerous skin lesion, is most common in older males with fair skin (Fitzpatrick Skin Phototypes I and II) and cumulative UV exposure [1–3]. Chronically photo-exposed areas such as the scalp, face, back of hands, and forearms are the most at risk [2]. Histologically, AK presents as dysplastic keratinocytes with enlarged hyperchromatic nuclei [1]. Lesions appear as red or brown rough, scaly patches, papules, or plaques in the stratum corneum layer of the epidermis, often with notable hyperkeratosis [1–3].

The clinical significance of AK is underscored by its potential to progress to squamous cell carcinoma (SCC), a type of non-melanoma skin cancer [2, 4–6]. Current treatments for AK are employed to prevent malignant transformation into SCC and resolve the aberrant appearance of lesions. Among the most common treatments are photodynamic therapy, cryotherapy, 5-fluorouracil (5-FU), imiquimod, trichloroacetic acid (TCA), use of ablative fractional laser, and combination therapy [2, 7, 8].

Accurate identification and diagnosis of AK is a complex and critical task, pivotal in preventing the development of SCC and establishing an effective treatment plan. Moreover, the evaluation of treatment effectiveness and the validation of existing and new treatment approaches heavily rely on their accurate diagnosis. However, the clinical variability of AK and the subjective nature of assessments present significant challenges, leading to the lack of a universally accepted standard for measuring AK.

This review evaluates the current methodologies and technologies used in evaluating and diagnosing AK. By analyzing clinician variability through inter- and intraobserver agreement in the characterization of AK, we seek to provide insights into how accurately the condition can be identified and quantified. Understanding the reliability of diagnostic tools from this perspective is imperative for medical professionals to make informed decisions regarding treatment options and monitoring strategies. This review also considers the transformative potential of a novel examination approach, offering insight into a future method that could improve the measurement and management of AK. Through a comprehensive synthesis of existing research, this review will enhance clinical practices and, ultimately, patient outcomes in AK management.

## Materials and methods

Studies published in peer-reviewed journals focusing on the measurement and diagnosis of AK in the context of clinician variability were comprehensively analyzed. The databases searched include PubMed, JAMA Network, and Google Scholar. The search was conducted in English, using keywords including ‘actinic keratosis,’ ‘metrics,’ reliability,’ and ‘measurement.’ Studies were selected based on their relevance, robustness of methodology, and specificity of their findings related to clinician accuracy and variability in measuring AK. Eight papers were identified. All evaluated interobserver agreement in AK examination and diagnosis, and three also examined intraobserver agreement.

## Results

### Overview of study characteristics

The reviewed literature consists of studies assessing interobserver and intraobserver agreement among physicians when measuring and evaluating AK. These studies span diverse geographic locations, clinical settings, and methodologies, highlighting the global importance of consistent AK measurement. Key characteristics such as sample size, number of physicians involved, and measurement tools used are summarized in Table 1.

**Table 1:** Summary of study characteristics

Study	Population Size	Number of Evaluators	Evaluation Method	Was there agreement?			
				Statistical Analysis	Interobserver	Intraobserver	Summary
Chen et al. [19]	9	12	Clinical categories and AK count categories	ICC	-0.06-0.54 pre-consensus meeting 0.12–0.66 post-consensus meeting	N/A	Poor to moderate interobserver agreement before and after meeting
Weinstock et al. [9]	9	7	Total lesion count	SD of Poisson regression parameter estimates	0.45 pre-consensus meeting 0.24 post-consensus meeting	N/A	Significant interobserver disagreement before and after meeting
Pellacani et al. [10]	30	4	Total lesion count and AKASI	ICC	0.92–0.94	N/A	Excellent interobserver agreement
Schmeusser et al. [11]	55	15	Photodamage evaluation with a 10-point scale	κ	0.114	0.473	Slight interobserver agreement Moderate intraobserver agreement
Zhu et al. [12]	17	67	Evaluation of Mohs frozen sections	κ	0.23	N/A	Fair interobserver agreement
Dréno et al. [13]	96	8	AK-FAS	κ	0.38–0.71	evaluated, no value provided	Fair to substantial interobserver agreement Moderate to near-perfect intraobserver agreement
Ianhaz et al. [14]	43	4	Total lesion count	ICC	0.74–0.77	0.30–0.93	Good interobserver agreement Poor to excellent intraobserver agreement
Tan et al. [15]	206	5	Teledermoscopy	κ	0.32–0.67	N/A	Moderate interobserver agreement

Abbreviations: AK - Actinic Keratosis; AKASI - Actinic Keratosis Area Severity Index; AK-FAS - Actinic Keratosis Field Assessment Scale; ICC - Intraclass Correlation Coefficient; κ - Kappa; SD - Standard Deviation

### Understanding statistical analysis methods

Research teams employed various statistical techniques to assess the reliability of AK characterization methods and the level of agreement between physician evaluators. Understanding these methods is crucial not only for interpreting study results but also for their practical application in the field of dermatology.

Kappa (κ) is a commonly used statistical measure to assess the agreement between raters for categorical variables [16]. It provides a nuanced evaluation by considering agreement due to chance [16]. The scale ranges from − 1 to 1, with higher values indicating agreement beyond chance, which is desirable for AK diagnosis [16]. The scale is as follows:

- < 0: Less than chance agreement.
- 0.01–0.20: Slight agreement.
- 0.21–0.40: Fair agreement.
- 0.41–0.60: Moderate agreement.
- 0.61–0.80: Substantial agreement.
- 0.81–1.00: Almost perfect agreement.

The Intraclass Correlation Coefficient (ICC), another measure of reliability, was used by research teams. The ICC has a range of 0 to 1, with higher values denoting more robust reliability [17]. The following scale is generally used:

- < 0.5: poor reliability.
- 0.5–0.75: moderate reliability.
- 0.75–0.9: good reliability.
- > 0.90: excellent reliability.

The standard deviation (SD) of estimates obtained from regression models was also utilized. SD measures the variability or spread of data from estimates [18]. In studies where dermatologists independently counted AK on patients, these estimates’ SDs help assess the counts’ consistency or reliability among different observers. A smaller standard deviation indicates less variability among estimates, meaning that there was better agreement among the observers, thereby suggesting a more reliable diagnostic tool.

### Interobserver agreement

Interobserver agreement measures the consistency among different physicians evaluating the same patients. The studies reviewed indicate significant variability in statistical parameter values, highlighting a critical need for better methods to achieve more reliable AK measurements. No study achieved perfect agreement.

1. Chen et al. conducted a study in 2013 involving twelve dermatologists and nine patients [19]. The research team evaluated the reliability of total lesion counts, lesion counts by size, total body surface area involving lesions, and total lesion counts of specific clinical presentations (e.g., “erythematous”) [19]. All counts were performed before and after a consensus discussion [19]. Statistical analyses used ICC evaluation. For the pre-meeting identification of lesions, values ranged from − 0.06 to 0.54, indicating poor-to-moderate reliability in AK characterization [19]. After consensus discussion, ICC values increased modestly to 0.12–0.66, still indicating poor to moderate agreement among dermatologists [19]. Note that the most reliable pre-consensus discussion method was counting lesions with hypertrophic characterization (ICC = 0.54), while the least reliable method was counting small lesions (ICC=-0.06) [19]. After the discussion, the most reliable method was total lesion count (ICC = 0.66), while the least reliable was the evaluation of total body surface area with lesions (ICC = 0.12) [19].
2. Weinstock et al. assessed the reliability of counting the total number of AK lesions in 2001 [9]. Seven dermatologists examined nine patients [9]. Observers completed evaluations before and after a consensus discussion [9]. The standard deviation of Poisson regression parameter estimates was used for statistical analyses. There was substantial variation in the reported number of AK lesions among dermatologists (SD = 0.45), which improved modestly to 0.24 after the consensus discussion [9]. Given that despite the consensus discussion, considerable variation remained, this study emphasizes that counting the total number of AK lesions is an unreliable diagnostic method.
3. Pellacani et al. conducted a pilot study in 2018 that evaluated the reproducibility of the Actinic Keratosis Area Severity Index (AKASI) and compared it with the method of total lesion count for assessing AK severity [10]. Four dermatologists evaluated thirty patients [10]. The AKASI is a novel tool that quantifies AK severity by considering the total skin involvement and severity of clinical signs of AK on a scale of 0–18 [20]. This study employed ICC evaluation for statistical analyses and indicated a high level of agreement between dermatologists in the characterization of AK lesions (ICC = 0.92–0.94) [10]. However, remember that this was a pilot study involving thirty patients and only four dermatologists, highlighting the need for further research on the reliability of the AKASI.
4. Schmeusser et al. investigated the inter-physician variation in quantifying skin photodamage of forearms using a 10-point Global Assessment Severity Scale for the evaluation of AK in 2020 [11]. Fifteen dermatologists evaluated the forearms of fifty-five patients [11]. Statistical analyses utilized κ. There was only sight interobserver agreement (κ = 0.114), indicating that photodamage evaluation is a poor metric for the characterization of AK lesions with low reliability [11].
5. Zhu et al. in 2023 examined how consistently sixty-seven Mohs surgeons and dermatopathologists could differentiate between AK and squamous cell carcinoma in situ (SCCis) when reviewing Mohs histological frozen Sect [12]. This study is particularly interesting, as it focused on the agreement among pathologists or dermatologists in making distinctions between AK and SCCis, which is critical for accurate diagnosis and treatment during Mohs micrographic surgery [12]. κ was utilized for statistical analyses. Results indicated fair interobserver agreement (κ = 0.23) in the identification of AKs [12]. Given this low level of agreement between raters, it is clear that there is notable variability among Mohs surgeons in interpreting the spectrum of AK.
6. Dréno et al. evaluated interobserver agreement when a recently developed diagnostic system, the Actinic Keratosis Field Assessment Scale (AK-FAS), in 2017 [13]. Eight examiners utilized the tool to evaluate AK in ninety-six patient photographs [13]. With the AK-FAS, clinicians review and rate photographs based on three criteria: total skin area affected by AK, hyperkeratosis, and sun damage to assess AK severity [13]. Statistical analyses utilized κ. Interrater κ values ranged from 0.38 to 0.71 between AK-FAS categories, indicating fair to substantial agreement between examiners [13]. Note that the investigators who developed the AK-FAS achieved the higher κ scores that suggest moderate to substantial interobserver agreement [13]. In contrast, the untrained investigators who validated the tool only achieved fair to moderate agreement in their characterization of AK [13].
7. Ianhaz et al. assessed the reliability of total lesion counts in diagnosing and examining AK on the face and forearms in 2013 [14]. Four dermatologists evaluated forty-three patients [14]. Statistical analyses used ICC evaluation. The study team found that overall, facial and forearm AK interobserver ICC values were 0.74 and 0.77, respectively [14]. Such values indicate good reliability of total lesion counts but not perfect agreement between raters [14]. The research team highlights that more variation existed with an increased population of raters and number of AK lesions, and the method thus became less reliable [14]. Therefore, the overall reliability of counting the total lesions remains questionable.
8. Tan et al. studied the interobserver diagnostic agreement in teledermoscopy in 2010 [15]. Five dermatologists viewed two hundred and six patient photos, and lesions were classified and diagnosed as various benign and malignant skin lesions, including AK [15]. Statistical analyses were performed using κ evaluation. Interobserver agreement between dermatologists was moderate (κ = 0.32–0.67) [15].

### Intraobserver agreement

Intraobserver agreement measures the consistency of the same physician’s evaluation or diagnosis of AK over time. Three of the aforementioned studies additionally evaluated this metric. Intraobserver agreement generally demonstrated better reliability than interobserver agreement, but perfect consistency was still not achieved.

1. Schmeusser et al. investigated the intraobserver variation as well in their study quantifying skin photodamage of forearms using the 10-point Global Assessment Severity Scale [11]. Statistical analyses similarly utilized κ. There was only moderate intraobserver agreement (κ = 0.473), indicating that photodamage evaluation is a poor marker in the characterization of AK lesions with low reliability [11].
2. Dréno et al. evaluated intraobserver agreement when examiners utilized AK-FAS multiple times on the same photographs [13]. Just as for interobserver analyses, statistical analyses utilized κ evaluation. However, no κ values were provided. The investigators reported that intraobserver agreement ranged from moderate to near perfect for all investigators across examinations [13].
3. Lanhaz et al. evaluated the reliability of total lesion counts in diagnosing and examining AK by the same raters over time [14]. Just as for interobserver agreement, statistical analyses were performed using ICC analysis. The study team found that facial and forearm AK intraobserver ICC values ranged from 0.30 to 0.93 [14]. This indicates poor to excellent reliability of total lesion counts but not perfect agreement between the same raters over time [14]. The most variation existed with increased lesion number, further questioning the overall reliability of counting the total lesions [14].

## Discussion

The reviewed studies underscore the crucial role of medical professionals in diagnosing and measuring AK accurately with reliable methods to evaluate the clinical presentation of lesions. The studies highlight substantial variability in interobserver and intraobserver agreement, with many methods showing only slight to moderate reliability. Given that AK is a precancerous condition, inconsistent diagnoses can lead to consequential treatment discrepancies and potentially increase the risk of malignant transformation. The modest improvements seen with consensus discussions and simplified rating scales underscore the need for more robust, standardized diagnostic methods. A limitation of this analysis is that only eight studies were identified for assessment. Further research is needed to understand the reliability of current methods for evaluating and diagnosing AK and develop better standardized, reproducible methods.

Several factors can influence the variation evidenced by the studies analyzed in this literature review, and understanding these factors is critical to improving clinical practice.

1. Experience and Training: The level of experience and training of the observers can significantly affect their ability to diagnose AKs accurately. More experienced dermatologists may have better diagnostic accuracy due to their familiarity with the clinical presentation and various subtypes of AK [21].
2. Clinical Presentation: Variability in the clinical presentation of AKs can contribute to differences in diagnosis among observers. AKs can manifest in various forms which may be subtle and easily overlooked, especially in the early stages.
3. Skin Type and Phototype: Differences in skin type and phototype among patients can affect the appearance of AKs and make their diagnosis more challenging [2]. For example, AKs may be more difficult to detect in individuals with darker skin tones due to reduced contrast between the lesions and surrounding skin.
4. Use of Dermoscopy: Dermoscopy, a noninvasive technique for examining skin lesions using a handheld magnification device and polarized light, can improve diagnostic accuracy [2, 22]. Huerta-Brogeras et al. calculated the sensitivity and specificity of dermoscopy for the diagnosis of AK, finding a sensitivity of 98.7% and a specificity of 95.0% [23]. However, interpreting dermoscopic features requires training and experience, and variability in interpretation among observers can occur [22].
5. Histopathological Variability: In cases where a biopsy or histopathological examination is performed to confirm the diagnosis, histopathological interpretation among pathologists can influence intraobserver and interobserver agreement in AK diagnosis [12].
6. Diagnostic Criteria and Guidelines: Differences in the diagnostic criteria and guidelines used by observers can lead to variability in AK diagnosis. Consensus guidelines may help standardize diagnosis but still leave room for interpretation, especially in borderline cases.
7. Presence of Confounding Factors: Other skin conditions, such as seborrheic keratosis, psoriasis, and squamous cell carcinoma in situ, can mimic the clinical presentation of AKs, leading to misdiagnosis or variability among observers [24].
8. Environmental Factors: Environmental factors, such as lighting conditions during clinical examination, can affect the visibility and interpretation of skin lesions, potentially influencing intraobserver and interobserver agreement in AK diagnosis.

Developing and implementing better evaluation tools is crucial to achieving consistent, accurate AK assessments and improving patient care and management. Advancements promise more effective and precise treatments, offering hope for better outcomes for AK patients. We offer a novel solution that, though yet to be tested, may improve diagnostic reliability and minimize subjectivity in interpreting AK. The histological characteristics of AK provide an avenue for enhanced visualization methods. AK lesions appear as red or brown rough, scaly patches, papules, or plaques in the stratum corneum, the outermost layer of the epidermis [1–3]. Staining this layer could improve AK visualization and, thus, clinician identification. A promising stain candidate is 1,3-dihydroxyacetone (DHA), a commercially obtained three‐carbon sugar commonly used in sunless tanning products [25, 26]. DHA chemically reacts with lysine residues in the stratum corneum, the same epidermal layer as AK, to temporarily create pigment [2, 25, 26].

Topical application of DHA to sun-exposed regions of skin hours before dermatological evaluation for AK could mimic the pigmentation process and act as a noninvasive marker on the skin. This approach could make AK lesions more visible, dramatically reducing subjectivity and the risk of missed diagnosis. Further, this approach could enhance early detection and mitigate the threat of malignant transformation to SCC. Note that the accuracy of DHA staining in distinguishing AK from other hyperkeratotic conditions like psoriasis or seborrheic keratosis, as well as the intensity of DHA staining of lesions based on skin type, hydration level, and application technique, is unknown and should be evaluated before use in clinical practice.

To conclude, the variability observed among studies emphasizes the need for standardized diagnostic methods that enhance consistency across medical professionals. Addressing the factors contributing to clinician variability in the evaluation and diagnosis of AK through standardized training, diagnostic aids like dermoscopy, and adherence to consensus guidelines can help minimize intraobserver and interobserver variation. Implementing advanced visualization techniques, such as DHA staining, is a promising approach to mitigate subjectivity and improve early detection. However, further research is necessary to validate the techniques and address potential limitations before widespread clinical adoption. Ultimately, advancing diagnostic tools and methodologies will enhance patient outcomes and reduce the risk of malignant transformation in individuals with AK.

## Data Availability

No datasets were generated or analysed during the current study.

## Code availability

Code availability is not applicable to this review article.
